# SARS-CoV-2 seroconversions and chains of infection in healthcare professionals in a German maximum care provider (The CoSHeP study)

**DOI:** 10.1007/s15010-021-01641-6

**Published:** 2021-06-18

**Authors:** Kathrin van Bremen, Malte Monin, Anna Maria Eis-Hübinger, Benjamin Marx, Souhaib Aldabaggh, Hendrik Streeck, Jan-Christian Wasmuth, Tanja Menting, Stefan Schlabe, Gereon J. Rieke, Carolynne Schwarze-Zander, Jürgen K. Rockstroh, Christoph Boesecke

**Affiliations:** 1 Department of Medicine I, Bonn University Hospital, Venusberg-Campus 1, 53127 Bonn, Germany; 2 German Centre for Infection Research (DZIF), Partner-site Cologne-Bonn, Bonn, Germany; 3 Institute of Virology, Medical Faculty, University of Bonn, Bonn, Germany; 4 Occupational Health Service, Bonn University Hospital, Bonn, Germany

**Keywords:** SARS-CoV-2-IgG, Healthcare-professionals, Chains of infection, Persisting immunity

## Abstract

**Introduction:**

The CoSHeP study provides novel data on SARS-CoV-2 seroconversion rates in healthcare professionals (HP) at risk at the University Hospital Bonn, a maximum healthcare provider in a region of 900.000 inhabitants.

**Methods:**

Single-center, longitudinal observational study investigating rate of SARS-CoV-2 IgG seroconversion in HP at 2 time-points. SARS-CoV-2 IgG was measured with Roche Elecsys Anti-SARS-CoV-2 assay.

**Results:**

Overall, 150 HP were included. Median age was 35 (range: 19–68). Main operational areas were intensive care unit (53%, *n* = 80), emergency room (31%, *n* = 46), and infectious disease department (16%, *n* = 24). SARS-CoV-2-IgG was detected in 5 participants (3%) at inclusion in May/June 2020, and in another 11 participants at follow-up (December 2020/ January 2021). Of the 16 seropositive participants, 14 had already known their SARS-CoV-2 infection because they had performed a PCR-test previously triggered by symptoms. Trailing chains of infection by self-assessment, 31% (*n* = 5) of infections were acquired through private contacts, 25% (*n* = 4) most likely through semi-private contacts during work. 13% (*n* = 2) were assumed to result through contact with contagious patients, further trailing was unsuccessful in 31% (*n* = 5). All five participants positive for SARS-CoV-2 IgG at inclusion remained positive with a median of 7 months after infection.

**Discussion:**

Frontline HP caring for hospitalized patients with COVID-19 are at higher risk of SARS-CoV-2 infections. Noteworthy, based upon identified chains of infection most of the infections were acquired in private environment and semi-private contacts during work. The low rate of infection through infectious patients reveals that professional hygiene standards are effective in preventing SARS-CoV-2 infections in HP. Persisting SARS-CoV-2-IgG might indicate longer lasting immunity supporting prioritization of negative HP for vaccination.

## Introduction

With first cases of the novel severe acute respiratory syndrome coronavirus type 2 (SARS-CoV-2) reported in December 2019 in China, the virus causing corona virus disease-19 (COVID-19) has been arising to a global pandemic with more than 88 million cases so far and almost 2 million deaths worldwide [1]. Until January 10th 2021, Germany has reported 1.9 million cases and 40.343 deaths [2]. The Rhineland area in particular was hit by 20.437 infections with an incidence of 2.2% and 309 deaths.

Frontline healthcare professionals (HP) caring for hospitalized patients with COVID-19 might be at higher risk both for acquiring as well as for spreading SARS-CoV-2 infections to other HP and community members [3]. Protective equipment for HP treating suspected and diagnosed patients with COVID-19 include FFP2 masks, single-use gowns, safety glasses, gloves, and medical caps. The prevalence of SARS-CoV-2 antibody in HP varies from less than 2% in a German cohort during the first wave of COVID-19, 11.2% in a Spanish cohort during the first peak in spring 2020 to 19.1% in Sweden [4–6]. Data on SARS-CoV-2 seroprevalence in HP in Germany as well as the lasting of SARS-CoV-2-IgG are sparse. Above all, the persistence of SARS-CoV-2-IgG is of special interest as it might be interpreted as post-infection-immunity. The CoSHeP study provides novel data on SARS-CoV-2 seroconversion rates in HP at the University Hospital Bonn, a maximum healthcare provider in a region of more than 900.000 inhabitants. The University Hospital Bonn has treated 463 hospitalized COVID-19 patients so far, whereas the majority of patients were initially seen in the emergency room and later on treated in the infectious disease ward or the internal medicine intensive care unit.

To our knowledge, this is the first longitudinal German study investigating the rate of SARS-CoV-2-IgG-seroconversion in at risk HP at a maximum care provider.

## Methods

Single-center, longitudinal observational study investigating the rate of SARS-CoV-2 IgG seroconversions in HP at two time-points (May/June 2020 and December 2020/January 2021). All employees from emergency room, internal medicine intensive care unit, infectious diseases ward, and infectious diseases outpatient clinic were invited by e-mail and leaflets to participate in the study. Study inclusion took place between May 27th and June 30th 2020. There were no exclusion criteria. All participants signed informed consent, the study was approved by the local ethics committee Nr. 191/20 and conducted according to the declaration of Helsinki. Participants were asked to fill out a questionnaire concerning demographic data, profession (nurse, physician, assistant personnel), actual or prior symptoms (fever, cough, dyspnea, ageusia/anosmia, common cold or others), prior SARS-CoV-2 PCR-testing and result, qualitative and quantitative contact to suspected and diagnosed COVID-19 patients in professional and in private environment, travel history including travel to hotspots as declared by the RKI during the first wave in Germany, and number of contacts in private environment in last 28 days prior to enrolment as well as self-assessment whether or not having been infected with SARS-CoV-2. 7.5 ml serum was analyzed for underlying SARS-CoV-2-IgG in each participant at both time-points. Serologic testing of SARS-CoV-2-IgG was performed with Roche Elecsys Anti-SARS-CoV-2 assay. In this assay, a recombinant protein representing the viral nucleocapsid(N)-antigen is used. Specificity and sensitivity claims 99.5%/99.5% > 14 days after PCR confirmation in manufactures labeling.

Statistical analysis was performed with IBM SPSS 26. Fisher’s exact, chi-squared, and Mann–Whitney *U* test were used for statistical analysis.

## Results

Baseline characteristics are shown in Table 1. Of overall 186 HP working in frontline 150 HP (81%) participated at inclusion, 63% (*n* = 94) were female, 37% male (*n* = 56). Median age was 35 (range: 19–68). 53% (*n* = 80) worked on intensive care unit, 31% (*n* = 46) in emergency room, 9% (*n* = 14) on infectious diseases ward, 7% (*n* = 10) in infectious diseases outpatient clinic. 68% (*n* = 102) were nurses, 14% (*n* = 21) physician, and 18% (*n* = 27) were healthcare-assistant personnel (e.g., students, cleaners, or physician-assistants).

**Table 1:** Baseline characteristics

Demographic characteristics *n* = 150	
Age	35 (19–68)
Female	94 (63%)
Male	56 (37%)
Working area	
Intensive care unit	80 (53%)
Emergency room	46 (31%)
Infectious disease department	24 (16%)
Profession	
Nurse	102 (68%)
Physician	21 (14%)
Assistant personnel	27 (18%)
Symptoms since begin of pandemic	43 (28%)
Previous PCR-testing *n* = 148	
Yes	55% (81)
No	45% (67)
Positive	4% (3)

At inclusion, 28% (*n* = 43) had experienced symptoms since the beginning of the pandemic. The most common symptoms were cough (63%, *n* = 27), common cold (60%, *n* = 26), and fever (44%, *n* = 19). 61% (*n* = 91) had not experienced any symptoms so far. Of note, 11% (*n* = 16) did not provide data concerning symptoms. PCR-testing had been performed in 55% of participants (*n* = 81/148, two participants provided no information concerning previous PCR-testing) until study inclusion, three (4%) had turned out positive. At inclusion, participants reported to have treated a median of 44 (range 1–900) suspected and 9 (range 0–75) confirmed cases of COVID-19 patients. Private contact to suspected and diagnosed patients with COVID-19 occurred in 10% (*n* = 15/147) and 6% (*n* = 7/125) of participants, respectively. 3% (*n* = 4/146) had traveled to a risk-area as defined by RKI since February 2020.

78% (*n* = 113/145) doubted having underwent any infection with SARS-CoV-2. However, SARS-CoV-2-IgG was detected in five participants (3%; two physicians, three nurses) at inclusion. 60% (*n* = 3) had known their SARS-CoV-2-infection before. 60% (*n* = 3) of infected participants reported contact with suspected or diagnosed SARS-CoV-2-infected in private environment. One person (20%) had been in a high-risk region. 60% (*n* = 3) of the SARS-CoV-2-infected participants reported past symptoms during infection. All reported fever and cough, two of three (67%) additionally reported about dyspnea. Only one claimed anosmia and ageusia.

118 of the initial 150 HP participated in the follow-up in December 2020/ January 2021. 57% (*n* = 66/115) denied having had any symptoms after the first test period in May/ June 2020. 43% (*n* = 49/115) reported symptoms like common cold (45%, *n* = 22/49), cough (31, *n* = 15/49) or anosmia and ageusia (10%, *n* = 5/49). SARS-CoV-2 PCR-testing had been performed in 78% (*n* = 85/109) participants since inclusion, with a median number of three follow-up-tests (range 0–20). During the second period, a median of 74 (range 0–1000) suspected and 20 (range 1–100) diagnosed patients with COVID-19 had been treated by all participants. 22% (*n* = 24/111) reported contact with suspected and 9% (*n* = 9/105) with diagnosed SARS-CoV-2 infected people in private environment, respectively. 63% (*n* = 68/108) denied traveling between June and December 2020, while 37% (*n* = 40/108) reported vacation trips. Concerning private contacts, participants reported contacts with in median 9 (range 0–50) persons outside professional environment within last 28 days before current test. In own assessment, 80% (*n* = 86/108) doubted having undergone SARS-CoV-2-infection, while 20% (*n* = 22/108) presumed a past infection. SARS-CoV-2-IgG was detected in 16 participants during follow-up resulting in an overall prevalence of 14% (*n* = 16/118). In detail, at inclusion we found a prevalence of 3% (*n* = 5/150) and of 10% (*n* = 11/113) at follow-up. Thus, additionally to the five cases at time of inclusion, 11 new infections were detected during follow-up. SARS-CoV-2-IgG remained positive in all participants with detectable SARS-CoV-2-IgG from time of inclusion. All newly participants with detectable SARS-CoV-2-IgG had shown typical symptoms and positive SARS-CoV-2-PCR-testing before. Moreover, all participants with diagnosed SARS-CoV-2-infection at inclusion underwent further PCR-testing between June and December 2020 and all remained negative at any time.

Regarding both time-points (inclusion and follow-up), SARS-CoV-2-infections mostly occurred on the infectious disease ward (38%, *n* = 6) whereas 31% (*n* = 5) occurred on the intensive care unit, 25% (*n* = 4) in the emergency room and 6% (*n* = 1) in the outpatient clinic. Regarding professions, the prevalence was highest in nurses (68%, *n* = 11/16), followed by physicians (19%, *n* = 3/16) and 13% (*n* = 2/16) in medical assistant personnel. There was no statistically significant correlation between professions and seroconversion at both time-points (*p* = 0.168 at inclusion; *p* = 0,979 at follow-up). Trailing chains of infection was not possible in 31% (*n* = 5), whereas 31% (*n* = 5) most likely acquired infection during private contacts outside the hospital. Another 25% (*n* = 4) of infections were most likely due to semi-private contacts during work (e.g., locker rooms, breaks, smoking). 13% (*n* = 2) were probably caused in contact with contagious patients.

## Discussion

To our knowledge, this is the first longitudinal German study investigating the rate of SARS-CoV-2 IgG seroconversions in frontline HP at a maximum care provider.

As expected, the infection rate in frontline HP is directly linked to the general prevalence in the region where the hospital is located. The initial low prevalence of 3% in HP represents the low overall infection rate in the Rhineland region during the first wave with an overall prevalence in general population of 0.2%. With the beginning of the second wave in October 2020, the rate of infections in the Rhineland region was rising with rates of new infections above 200 per 100.000 inhabitants per week in December 2020 [2]. Thus, the number of patients with COVID-19 in hospital increased steadily. In the follow-up examination in December 2020/January 2021, the prevalence of SARS-CoV-2-IgG in HP had tripled.

Our data reveal that 31% of infections were acquired due to private contacts. We observed certain clusters of infection in people with close contact at work. Thus, we assume that another 25% of the infected participants acquired infection with SARS-CoV-2 during semi-private contacts at work (e.g., due to work in same shifts, using locker rooms at the same time and close contacts during breaks and in smoking areas). Direct contact to patients with COVID-19 was only responsible for 13% of all infections. Taking both groups together, one might assume that about 38% of all infections of frontline HP are linked to the working area. The origin of the missing 31% of infections could not be trailed. The overall low incidence of infections due to direct contact with patients indicates that the protective equipment for HP (FFP2 masks, single-use gowns, safety glasses, medical cap) seems to be effective in preventing infections as shown by the low rate of acquired infections via direct medical care. Considering the fact that the majority of infections were probably caused by private and/or semi-private contacts at work, adherence to general COVID-19 hygiene rules (distancing, hand disinfection, face masks and venting) also at the workplace even when not being involved in direct patient care needs to be significantly improved to prevent infections.

Several cohorts have documented the occupational risk of SARS-CoV-2 infection in HP. A recent cohort from Sweden confirmed 19.1% rate of seroprevalence in HP while cohorts from centers in the UK and Belgium showed seroprevalence rates of 11% and 7.6%, respectively [4, 7, 8]. In Germany, there has been reported a low rate of seroprevalence with 1.6% during the first wave [9]. Our higher rate resulted from different circumstances. The rate is calculated with the data from both the first and the second wave with a far higher overall prevalence. Additionally, our cohort has a positive selection bias as only HP were enrolled who have been working in one of the COVID-19 affected areas of the hospital. Frontline HP with contact to COVID-19-patients have been shown to be at higher risk for SARS-CoV-2 infection than HP with non-COVID-19 patient contact [4]. Since beginning of the pandemic, the University Hospital Bonn has reported 280 positive SARS-CoV-2 RT-PCR tests in 22.604 tests performed in employees from all sections of the hospital including employees without any patient contact, meaning a prevalence of 1.2%. One has to mention that some positive tests will be doubled due to repeating tests in employees before returning to work after quarantine, therefore, prevalence might be even lower underlining the higher risk of HP caring for COVID-19 patients.

Availability and correct use of protective equipment including FFP2 masks, single-use gowns, safety glasses, gloves, and medical caps are most important and effective in preventing infections. A reuse as well as inadequate use of protective equipment results in a higher risk of self-contamination. HP who reused protective equipment were found at highest risk of SARS-CoV-2 infection in a UK and US cohort [10]. Although all our participants had enough personal protective equipment and correct use was displayed at each patient room and in online tutorials, we cannot exclude incorrect usage or reusage.

Trailing chains of infection, most infections could be traced back to private contacts either in private environment or during semi-private contacts during work, e.g., in locker rooms, during breaks and while having close contact during smoking and not maintaining rules of distancing. 53% of participants had reported contact with more than five other members in the past 4 weeks. Our data surely highlight that private contacts must be reduced consequently, since infected employees might transmit infection to patients as well as to colleagues. Private contacts being most responsible for transmission in our cohort, might underline the good prevention via social distancing and protective equipment. Close contacts among employees during breaks, locker rooms or smoking might have triggered infections in our cohort particularly in the light that most of the infections occurred in a group of nurses on the infectious diseases ward.

An important prevention tool might be the low-threshold offer of routine SARS-CoV-2-PCR-testing in HP either with symptoms or asymptomatic. 55% of participants at inclusion and 78% of our participants at follow-up reported having undergone PCR-testing at work in the past. All but two of the positive SARS-CoV-2-IgG participants had shown positive PCR-testing before. With a rate of asymptomatic courses of 23% in a Spanish HP cohort, frequent testing of asymptomatic frontline HP seems valuable. Also, a large population-based study including HP in the US stated 92% of SARS-CoV-2 infected HP reporting at least one symptom as fever, dyspnea and cough [11]. In our cohort common cold was reported by 60% at inclusion and 45% of participants at follow-up, respectively. All but one of the SARS-CoV-2-IgG positive HP showed symptoms like cough, dyspnea, fever or anosmia/ageusia. Only one participant had to be hospitalized due to dyspnea. The mild course might be explained by the overall younger age of the infected as well as the lack of pre-existing illnesses.

Interestingly, all participants with positive SARS-CoV-2 IgG at inclusion remained IgG-positive at follow-up, with the longest duration being 9 months to follow-up. Of those initially IgG-positive tested participants all underwent further PCR-testing in between and remained PCR-negative. Thus, no re-infections were documented. The persistence of SARS-CoV-2-IgG and negative PCR-testing was also seen in a large cohort from Great Britain [12]. Persisting SARS-CoV-2 IgG may indicate protection from re-infection and has to be further evaluated. We have to state though that we did not perform further evaluation of neutralizing antibodies in those participants. Since the duration of immune protection has to be further assessed in larger cohorts with even longer follow-up particularly in the light of increases in mutations it remains important to test HP with symptoms even with past SARS-CoV-2 infection.

Our study has several limitations. The overall number of participants (150) was too low to allow for a multi-variate analysis. Some questionnaires missed certain answers leading to even lower baseline values. Moreover, some participants were lost to follow-up during the second period due to switch of operating areas and/or moving. One committed suicide. We would speculate that the fear of COVID-19 was rather decreasing which might have additionally led to less participation at follow-up. Due to the specific group of frontline HP caring for COVID-19 patients the SARS-CoV-2-IgG prevalence might be overestimated. Moreover, there is the possibility that antibody answer was too low to detect at sample point and SARS-CoV-2 infection had occurred without notice. Additionally, we did not measure specific spike-protein antibody. Nevertheless, our longitudinal study represents a real-life cohort of frontline HP caring for COVID-19 patients in Germany.

In conclusion, frontline HP are at higher risks of getting infected with SARS-CoV-2. With increasing numbers of SARS-CoV-2 infection in the general community numbers also increase among HP. Most infections were acquired in the private and semi-private environment. Social distancing also at the workplace has to be emphasized. Nonetheless, the data indicate that the correct use of protective equipment is effective in preventing infections.
